# Genetic diversities of *Mycobacterium tuberculosis* complex species in Western Kenya

**DOI:** 10.1099/acmi.0.000729.v3

**Published:** 2024-02-20

**Authors:** Charles Komen Chelimo, Paul Oyieng Angienda, Charles Ochieng Olwal, Henry Nyamogoba

**Affiliations:** ^1^​ Department of Zoology, Maseno University, Maseno, Kenya; ^2^​ Department of Microbiology, Moi University, Eldoret, Kenya

**Keywords:** genetic diversity, Hunter–Gaston diversity index (HGDI), *Mycobacterium* interspersed repetitive units (MIRU), *Mycobacterium tuberculosis* complex (MTBC), variable-number tandem repeats (VNTRs)

## Abstract

**Background.:**

Tuberculosis (TB) remains a high-burden infectious disease worldwide. *Mycobacterium tuberculosis* complex (MTBC) is the aetiological agent of TB.

**Research Gap.:**

The TB burden is significantly linked to the development of drug-resistant strains. Thus, there is an urgent need for close surveillance of MTBC circulating in a given region, such as Western Kenya, for treatment of TB.

**Aim.:**

To determine the proportion of MTBC species, strains and genetic diversities in circulation in HIV/AIDS-prevalent regions, and Western Kenya in particular. The clinical MTBC isolates were collected from Moi Teaching and Referral Hospital (MTRH) at Eldoret-Kenya during 2013–14. All clinical MTBC isolates were confirmed by the gold standard method (Löwenstein–Jensen medium culture) before inclusion in the investigation.

**Methodology.:**

Twelve-loci mycobacterium interspersed repetitive unit – variable-number tandem repeats (MIRU-VNTR) genotyping was performed to determine the circulating species/strains of MTBC using the www.miru-vntrplus.org web platform. Allelic diversity was calculated using the Hunter–Gaston diversity index (HGDI).

**Results.:**

The species *M. tuberculosis*, *Mycobacterium bovis*, *Mycobacterium africanum*, *Mycobacterium pinnipedii*, *Mycobacterium microti*, *Mycobacterium caprae* and *Mycobacterium canetti* were identified in the MTBC population. These strains were found in the Beijing, Latin American Mediterranean, Uganda 1/2, East African Indian, Ilama, West African 1/2, Harlem, URAL, Ghana, Seal, Cameroon and Vole etc. regions of Western Kenya. Notably, some isolates had unknown (new/unassigned) species. The strains were grouped into nine clusters with a clustering rate of 31.18 % and a high allelic diversity index of 0.53 was observed.

**Conclusion.:**

The present findings suggest that there is an urgent need for more awareness among healthcare professionals and stakeholders concerning the existence of foreign MTBC species/strains in Kenya. Furthermore, 12-loci MIRU-VNTR may not be suitable for the surveillance of MTBC strains in circulation in Kenya. Thus, high-resolution techniques such as whole-genome sequencing need to be adopted to resolve the genetic diversity and establish evolutionary trends for future and archived samples. This knowledge will be crucial in restraining TB, providing insights into new drug development, and developing prevention, control and treatment strategies for TB.

## Data Summary

The raw data have been uploaded as Supplementary Files, available in the online version of this article.

## Introduction

In 2021, approximately 10.6 million individuals contracted tuberculosis (TB), a slight increase from the 10.1 million cases reported in 2020. Additionally, the number of TB-related deaths in 2021 was ~1.6 million, including 187 000 people with HIV/AIDS, compared to 1.5 million deaths in 2020, which included 214 000 individuals with HIV/AIDS [1]. Furthermore, there was a 3.6 % rise in the tuberculosis incidence rate in 2021 compared to 2020, marking a departure from the nearly 2 % annual decrease observed over the past two decades [1]. Moreover, approximately 23 % of the world’s population has latent TB infection, and could develop active TB in their lifetime [2]. The African region plays a pivotal role in the global TB epidemiological context [3]. Kenya is a high-TB-prevalence country and reports substantial TB-related mortality [4].

TB represents a significant global public health issue, surpassing even HIV/AIDS in its impact. The diagnosis of TB poses considerable challenges due to the paucibacillary nature of specimens and the localization of the disease at anatomical sites that are challenging to access [5, 6]. Prior to the onset of the coronavirus disease 2019 (COVID-19) pandemic, TB stood as the primary cause of mortality attributed to a single infectious agent. However, in the current context TB has become the second leading infectious cause of death, following closely behind COVID-19 [7].

*Mycobacterium tuberculosis* is the main causative agent for TB [8]. There are eight species within the *M. tuberculosis complex* (MTBC), namely *M. tuberculosis*, *Mycobacterium bovis*, *Mycobacterium africanum*, bacillus Calmette–Guérin (BCG), *Mycobacterium microti, Mycobacterium caprae, Mycobacterium pinnipedii* and *Mycobacterium canetti* [9]. Pulmonary TB is the most common form of tuberculosis and primarily affects the lungs, often characterized by a persistent cough, chest pain, haemoptysis (coughing up blood) and respiratory symptoms. In contrast to pulmonary TB, extra-pulmonary TB refers to the infection of organs and tissues outside the lungs. This form of TB can affect various parts of the body, including the lymph nodes, bones, joints, meninges (lining of the brain and spinal cord), genitourinary system and other organs. Extra-pulmonary TB often occurs in individuals with weakened immune systems, but it can also affect immunocompetent individuals [5]. Although most pulmonary tuberculosis is mainly attributed to *M. tuberculosis,* there is need for surveillance of species and strains of MTBC in circulation for information on the use suitable drugs to minimize the development of anti-TB drug resistance [10].

Nontuberculous mycobacteria (NTM) are a diverse group of bacteria within the genus *Mycobacterium*, distinct from the tuberculosis complex. Unlike *M. tuberculosis*, NTM are opportunistic pathogens commonly found in environmental reservoirs such as soil and water. While generally posing a lower risk to healthy individuals, NTM infections can be problematic for those with compromised immune systems or respiratory conditions. NTM can lead to various infections, including pulmonary disease, lymphadenitis and skin infections. Due to species variability and diverse clinical presentations, diagnosing and treating NTM infections can be challenging [11].

The pathogenicity of MTBC is facilitated by various proteins, including early secretory antigenic target 6 (ESAT-6), which is present in both *M. tuberculosis* and *M. bovis* BCG. It plays a pivotal role in the virulence of these mycobacteria by contributing to the evasion of host immune responses and facilitating cell invasion, as highlighted by Kamra *et al*. [7]. Another noteworthy protein is the antigen 85 complex (Ag85), which is conserved across various mycobacterial species, including the MTBC and some non-tuberculous mycobacteria (NTM). This protein is actively involved in the biosynthesis of mycolic acids, crucial components of the mycobacterial cell wall, as elucidated by Khan *et al*. [7].

Comparative genome analyses have revealed several regions of difference (RDs) among mycobacterial species. RD4, which encompasses Rv1506c–Rv1516c of *Mycobacterium. tuberculosis* (*M. tb*) H37Rv, is absent in the closely related *M. bovis*, *M. bovis* BCG and NTM. The RD1 region encodes important virulence factors, including ESAT-6 and culture filtrate protein 10 (CFP-10). *M*. *bovis* and some BCG sub-strains lack the RD1 region [12, 13].

The emergence of multidrug-resistant (MDR) strains of *Mycobacterium* species has limited the available treatment options for tuberculosis, creating a risk for an untreatable and fatal disease [14]. Due the emergence of drug resistance, there have been reports on the species and strains circulating in some Asian and African countries [15–18]. A study reported on MDR *M. tuberculosis* genotypes in Nairobi, Kenya [19]. Nonetheless, this previous study only focused on mutations associated with MDR in *M. tuberculosis* genotypes. There is paucity of current data on the proportion of species and strains of MTBC circulating in the Western Kenya region. Epidemiologically, Western Kenya is relevant because it is a high-HIV-burden area [20], and hence bears the greatest burden of TB in Kenya [21]. Moreover, it is the gateway to and from other East African countries and can easily acquire strains from neighbouring countries.

Molecular epidemiology studies are used to identify factors affecting TB distribution and transmission. This is often done using molecular characterization techniques, such as spoligotyping and mycobacterium interspersed repetitive unit – variable-number tandem repeats (MIRU-VNTR) of genetic elements [22]. Genetic lineages of *M. tuberculosis* strains have previously been determined based on spoligotyping and whole-genome sequencing [18, 23]. MIRU-VNTR can determine the diversity and clonal expansion of strains at high resolution [24].

Ending the TB epidemic by 2030 is one of the United Nations Sustainable Development health targets. A better understanding of the genetic diversity of clinical isolates circulating in a country or region is essential in the fight against TB. Hence, this study aimed to determine the proportion of species and strains and the genetic diversity of MTBC circulating in Western Kenya from archived samples between the period 2013 and 2014 before the COVID-19 pandemic.

## Methods

### Clinical isolates and study design

The study was carried out at the Moi Teaching and Referral Hospital (MTRH), Eldoret town (0.5143 N, 35.2698 E), Kenya. MTRH is primary academic hospital serving the entire Western Kenya community [25].

This was a retrospective study in which samples archived in 2013 and 2014 were used. Only TB-positive samples based on Löwenstein–Jensen medium cultures were used. Forty archived samples positive for TB and without contaminants were used in this study. To eliminate contamination and increase the reliability and validity of our results, negative controls or blank samples, devoid of clinical material, were integrated into each step of the laboratory workflow, from DNA extraction to PCR amplification. These controls consisted of reagents without any DNA template, allowing for the detection of potential contamination introduced during laboratory procedures. Separate workstations were also dedicated for pre-PCR and post-PCR activities to prevent cross-contamination. UV irradiation of equipment and reagents was also performed regularly to eliminate potential contamination. The study was reviewed and approved by the Institutional of Research and Ethics Committee (IREC) of Moi Teaching and Referral Hospital (MTRH) and Moi University (approval number: IREC: 0001214). Permission to use the archived samples was also granted by the MTRH management. An informed consent waiver was granted because this study used archived samples.

### Sample processing

After confirming patient identification numbers from the laboratory records, corresponding archived TB-positive samples (stored at −80 °C), classified based on growth on Löwenstein–Jensen medium cultures, indicating the presence of *M. tuberculosis* complex, were retrieved and thawed at room temperature. Samples were purposively collected from Moi Teaching and Referral Hospital in Western Kenya. Upon collection, samples were immediately transported to the laboratory in a cold chain to ensure sample integrity. Samples with incomplete or inadequate information, as well as those with signs of degradation, were excluded.

### DNA extraction and dilution

Mycobacterial colonies that had grown on Löwenstein–Jensen were resuspended in a screwed cap tube. Subsequently, they were incubated at 95 °C for 45 min, utilizing a PCR cycler with a hot lid. The suspension underwent centrifugation at 15 000 ***g*** for 1 min to pellet the cell debris. The supernatant, which contained the DNA, was harvested and transferred into a new tube. The concentrated stocks were stored at –20 °C until they were utilized in subsequent procedures.

### MIRU-VNTR typing and allelic diversity determination

In this study, MIRU-VNTR typing with a panel of 12 loci was employed. Each locus underwent PCR amplification, following established procedures as previously outlined [26] using the primers provided in Table S1. Subsequently, the resulting products were subjected to electrophoresis on 2 % agarose gels, employing extended runs lasting 4 h. A 50 bp ladder was utilized as a reference for determining band sizes. The MIRU-VNTR bands corresponding to each locus were interpreted from the gel images, relying on reference table indications to determine the respective copy numbers. The species and strains were identified in the MIRU-VNTR*plus* database accessible at http://www.miru-vntrplus.org.

A neighbour-joining dendrogram was generated using MIRU-VNTR data (Table S2) through the implementation of the neighbour-joining algorithm. The algorithm iteratively merged the closest neighbours until a complete tree structure was formed. Clades or clusters were subsequently identified among isolates based on the distinctive branching patterns observed in the dendrogram. A cluster was defined as group of two or more strains with identical profiles. In addition, a minimum spanning tree was constructed to identify clonal complexes, which are defined as groups of isolates that are within dual-locus variants of each other.

The calculation for the discriminatory index [Hunter–Gaston diversity index (HGDI)] was based on Simpson’s index of diversity [15]. HGDI was used to quantify the discriminatory index and to evaluate the allelic diversity of the different MIRU-VNTR loci. Based on the respective HGDI scores, the discriminatory powers were classified as high (HGDI ≥0.6), moderate (0.3≤HGDI≤0.6) or low (HGDI ≤0.3) [27].

## Results

### Participants’ characteristics

In the present study, 40 archived samples from 57.5 and 41.7 % males and females, respectively, were used. The participants’ age ranged from 18 to 65 years with median age being 30 years.

### Clinical *M*. *tuberculosis* species and MTBC strains in circulation

From the 42 isolates, 3 *Mycobacterium* species, namely *M. tuberculosis*, *M. bovis* and *M. africanum*, *M. pinnipedii*, *M. microti*, *M. caprae* and *M. canetti* were identified, with *M. tuberculosis* being the most common (Table S3).

**Fig. 1.**
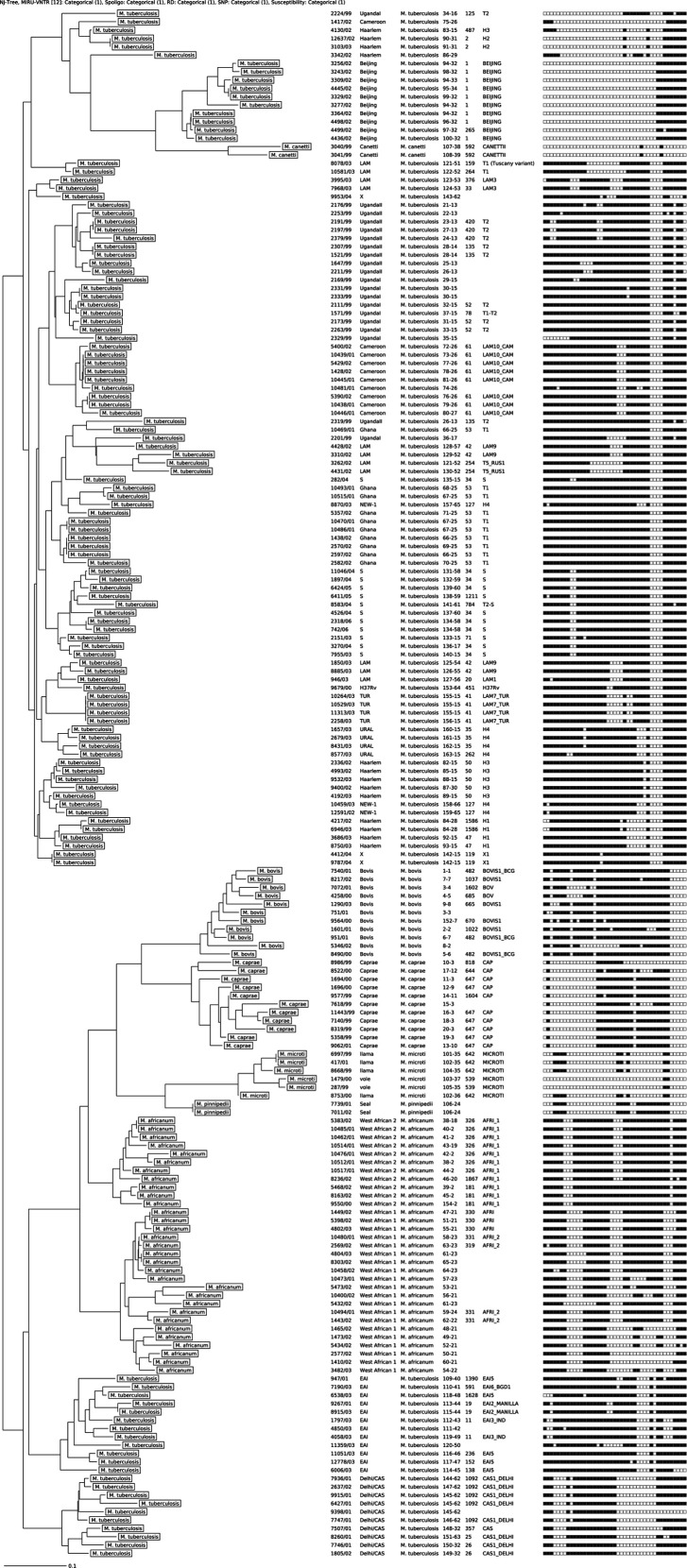
MIRU-VNTR dendrogram (12 loci) of the 42 *M. tuberculosis* clinical isolates analysed in the present study. The isolates were circulating in 2013 and 2014 in Western Kenya.

The study revealed that there were a number of MTBC strains circulating in Western Kenya. These strains included Beijing, Latin American Mediterranean (LAM), Uganda 1 and 2, East African Indian (EAI), Ilama, West African 1 and 2, Haelem, URAL, Ghana, Seal, Cameroon, Vole, etc. There were also combinations of Delhi/CAS strains of *M. tuberculosis*. Moreover, some strains were identified as s, X and NEW (Fig. 1).

### Cluster analysis of clinical isolates of MTBC

**Fig. 2.**
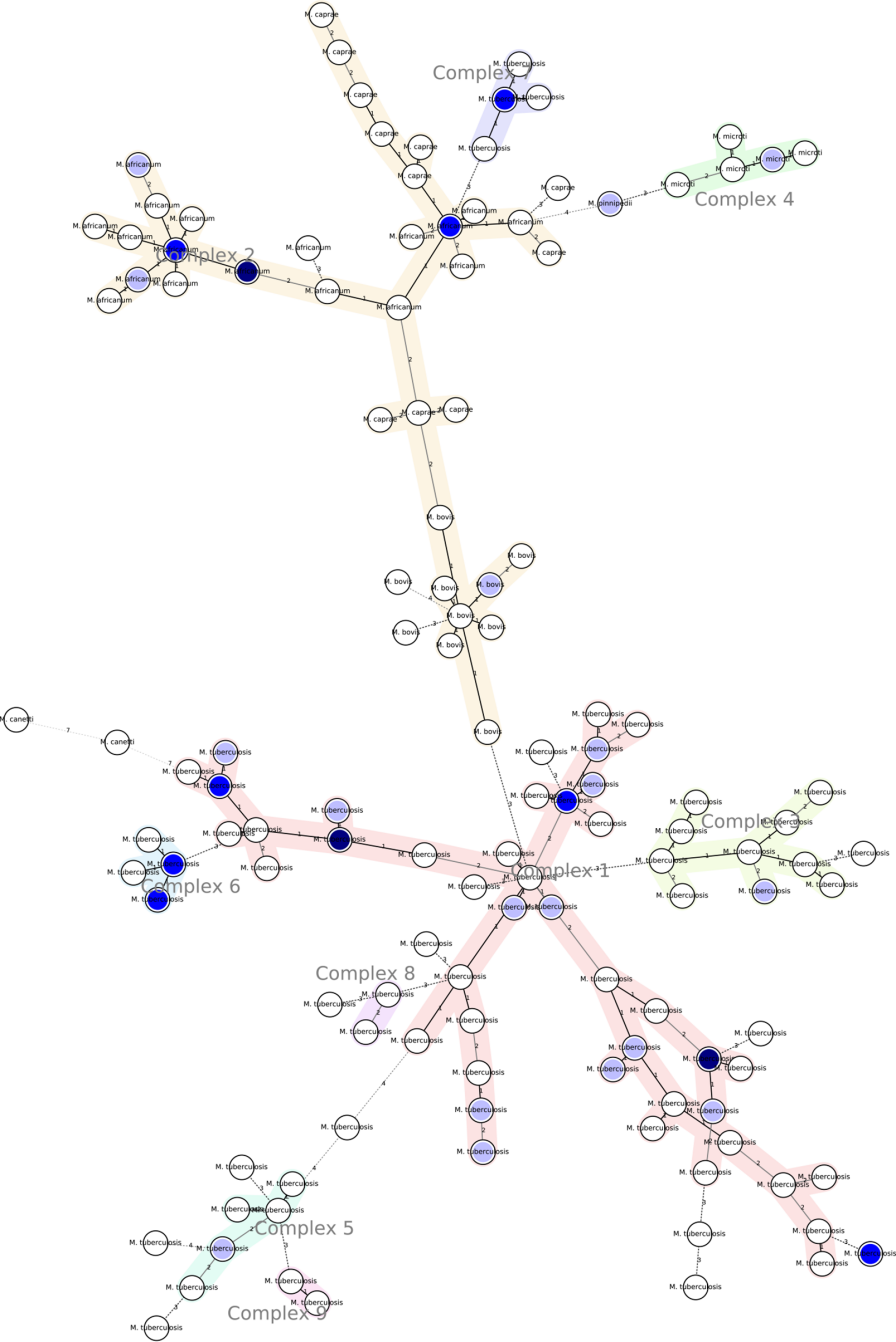
Minimum spanning tree of *M. tuberculosis* clinical isolates circulating in Western Kenya. The tree was constructed based on 12-loci MIRU-VNTR genotypic data.

A neighbour-joining dendrogram and a minimum spanning tree were generated for cluster analysis. Clustering rates of 31.18 and 50 % were observed for the MIRU-VNTR and Spoligo, respectively. Several clusters with varying numbers of strains were identified, as shown in Fig. 1. Furthermore, a minimum spanning tree showed that the known *Mycobacterial* isolates circulating in Western Kenya belong to nine clonal complexes (Fig. 2).

### Allelic diversity of clinical isolates of MTBC

The allelic diversity was calculated for each MIRU-VNTR locus. The allelic diversity index of *Mycobacterium* strains circulating in Western Kenya ranged from 0.17 to 0.76 with a mean HGDI of 0.53 (Table 1).

**Table 1.** Allelic diversity of MTBC circulating in Western Kenya between 2013 and 2014

Locus	HGDI
MIRU39	0.33
MIRU26	0.72
MIRU40	0.75
MIRU10	0.76
MIRU16	0.64
MIRU27	0.31
MIRU23	0.63
MIRU31	0.73
MIRU20	0.26
MIRU02	0.17
MIRU24	0.49
MIRU04	0.51

Note: High (HGDI≥0.6), moderate (0.3≤HGDI≤0.6) or low (HGDI≤0.3).

## Discussion

TB remains a major source of morbidity and mortality globally [1]. Thus, better ways of treating and controlling the spread of TB are imperative. This requires a better understanding of the MTBC species and strains circulating in a given region.

The present study showed that the main species found circulating Western Kenya in the region included *M. tuberculosis*, *M. bovis* and *M. africanum*. Further, there were unknown *Mycobacterium* species and multiple matches of *Mycobacterium* species mainly drawn from *M. tuberculosis* and *M. africanum*, with *M. tuberculosis* being the predominant species. The findings of the current study are in agreement with previous studies in Uganda [28], Tanzania [18] and Mozambique [17], in which *M. tuberculosis* was reported to be the most common circulating species. However, the findings are inconsistent with those of a previous study in which *M. africanum* was reported to be the most prevalent MTBC in Ghana [29]. The discrepancy between the present and the previous report with reference to *M. africanum* prevalence could be attributed to limited migrations and spread of *M. africanum*, which is predominantly located in West African countries [30]. The observation of different *Mycobacterium* species in circulation and some unknown species is a worrying trend because Western Kenya is gateway to most East African countries and could enhance the spread and development of genetic variants of *Mycobacterium*, which might render treatment difficult.

In the present study, several *Mycobacterium* strains were identified among the clinical isolates in circulation in Western Kenya. These strains included Beijing, LAM, Uganda I, Ural, EAI and multiple *M. tuberculosis* strains. Beijing and unknown were the predominant strains in the region. The present study is consistent with previous studies that have identified the Beijing family as the predominant strain in most countries [23, 31]. It is well established that the Beijing strain is most prevalent in the East Asian region, accounting for >50 % of all strains [32]. The high prevalence of Beijing in Western Kenya and in many other continents could be related to the high human migratory activities from and to East Asia. This view is supported by a study in Tanzania in which the Beijing strain was high in the Serengeti ecosystem, a popular tourist destination [31]. The LAM and EAI strains are commonly found in African countries that share a border with Mozambique, such as Tanzania, Malawi, Zambia and Zimbabwe [17]. The substantial economic activity between Kenya and many other countries in eastern and central Africa might be enhancing the transmission of LAM and EAI strains. The same explanation could be responsible for the presence of the Uganda I strain found in this study.

In agreement with a study in the East African region [31], the present study revealed a considerable prevalence of unknown *Mycobacterium* strains. Although the explanation for the existence of unknown *Mycobacterium* strains in Western Kenya remains unclear, a possible explanation could be related to epidemiological or evolutionary dynamics resulting in the formation of the unknown strains of the *Mycobacterium* in this region. An alternative explanation for the high prevalence of unknown strains could be related to the high burden of HIV in Western Kenya resulting in a large number of immune-compromised persons [20, 33], who facilitate evolution of bacterial pathogens [34], including *Mycobacterium* species.

MIRU-VNTR cluster analysis grouped the strains into several clusters, resulting in a fairly low clustering rate of 31.18 %. The clusters obtained in included Beijing, LAM, Ugandan I, Ural and EAI, among others. The clustering rate of 31.18 % observed in this study differs from those reported in three Asian countries [35] and other countries, such as Canada (75.69 %), PR China (18.4 %), Iraq (18.03 %), Tanga-Tanzania (21.3 %) and Taiwan, ROC (40.85 %) [35–39]. This discrepancy could be attributed to different assay methods, e.g. spoligotyping or 24- and 15-loci MIRU-VNTR. The low clustering rate observed suggests that there is a considerable degree of transmission of *Mycobacterium* strains in Western Kenya.

The MIRU-VNTR loci were found to be highly discriminatory (mean HGDI of 0.66) in Western Kenya. This was comparable to other previous studies in Canada [37], PR China [38], Iraq [36], Tanzania [39], Angola [40] and some Asian countries [35]. The moderate discriminatory power found in this study suggests that 12 MIRU-VNTR loci alone may not give an acceptable level of resolution for classifying MTBC circulating in a given region.

MIRU-VNTR has limited discriminatory power in certain settings, especially in regions with a high prevalence of specific strains, as in the case of Beijing. This means that it might not be able to differentiate between closely related strains, reducing its effectiveness in identifying unique sources of infection. Thus, the effectiveness of MIRU-VNTR can vary geographically, as different regions may have unique strain distributions [41]. The loci chosen for MIRU-VNTR analysis may not be equally informative in all settings, affecting its utility in certain regions. MIRU-VNTR markers may also exhibit homoplasy, i.e. the occurrence of identical patterns in unrelated strains, leading to potential misinterpretation of strain relatedness. This can be a challenge in accurately tracing the transmission of TB in a population. Moreover, some MIRU-VNTR loci may not be evolutionarily stable, and mutations can occur relatively frequently. This instability can affect the accuracy of the analysis, making it challenging to establish a clear evolutionary relationship between strains [42].

Despite its limitations, MIRU-VNTR remains a valuable tool for identifying clusters of TB cases and conducting contact tracing. Integrating MIRU-VNTR data with epidemiological information allows for a more comprehensive understanding of transmission dynamics and aids in targeted interventions during outbreaks. MIRU-VNTR analysis can be used in conjunction with drug susceptibility testing to monitor the spread of drug-resistant strains. This information is crucial for adapting treatment regimens and controlling the emergence of drug-resistant TB. The limitations of MIRU-VNTR highlight the importance of employing a multi-faceted approach to TB control. Public health interventions should not solely rely on molecular typing methods but should also incorporate clinical and epidemiological data to develop effective control strategies.

In summary, the findings align with research conducted in Canada, PR China, Iraq, Tanzania and Angola. Nevertheless, what sets these results apart is their distinctiveness, given that the study sample was sourced from Western Kenya, a region marked by elevated HIV prevalence. The epidemiological profile of this population is exceptional, featuring a noteworthy prevalence of HIV. Consequently, the genetic diversity observed in this context stands out as unique. The present study showed that there is more than one species of *M. tuberculosis* complex circulating in Western Kenya. Western Kenya has a number of *M. tuberculosis* strains in circulation, with the Beijing strain being predominant. The strains isolated in Western Kenya belong to different clusters and clonal complexes, with a moderate allelic HGDI suggesting that there is considerable genetic diversity among the MTBC circulating in the region. The HGDI of MIRU-VNTR was moderate, suggesting that 12-loci MIRU-VINTR alone may not be suitable for monitoring MTBC transmission in a region. Further studies based on superior approaches, such as combining MIRU-VNTR with spoligotyping or whole-genome sequencing, are needed to identify the unknown species and the susceptibility patterns of strains circulating in Western Kenya for better management of TB.

## Supplementary Data

Supplementary material 1
