# Supplementary material for: Genetic diversities of Mycobacterium tuberculosis complex species in Western Kenya
Source: Access Microbiol. 2024 Feb 20;6(2):000729.v3. doi: 10.1099/acmi.0.000729.v3 (PMC10928392; doi:10.1099/acmi.0.000729.v3)
Supplement: Supplementary material 1 [file acmi-6-729.v3-s001.pdf]

Table S1. Fluorescent primers for 12 loci MIRU-VNTR.

Locus	Alias	Repeat unit length, bp	PCR primer pair(5' to 3'), with labeling indicated
580	MIRU 4	77	GCGCGAGAGCCCGAACTGC (FAM)<br>GCGCAGCAGAAACGCCAGC
2996	MIRU 26	51	TAGGTCTACCGTCGAAATCTGTGAC<br>CATAGGCGACCAGGCGAATAG (VIC)
802	MIRU 40	54	GGGTTGCTGGATGACAACGTGT(NED)<br>GGGTGATCTCGGCGAAATCAGATA
960	MIRU 10	53	GTTCTTGACCAACTGCAGTCGTCC<br>GCCACCTTGGTGATCAGCTACCT (FAM)
1644	MIRU 16	53	TCGGTGATCGGGTCCAGTCCAAGTA<br>CCCGTCGTGCAGCCCTGGTAC (VIC)
3192	MIRU 31	53	ACTGATTGGCTTCATACGGCTTTA<br>GTGCCGACGTGGTCTTGAT (NED)
154	MIRU 02	53	TGGACTTGCAGCAATGGACCAACT<br>TACTCGGACGCCGGCTCAAAAT (FAM)
2531	MIRU 23	53	CTGTCGATGGCCGCAACAAAACG (VIC)<br>AGCTCAACGGGTTCGCCCTTTTGTC
4348	MIRU 39	53	CGCATCGACAAACTGGAGCCAAAC<br>CGGAAACGTCTACGCCCCACACAT (NED)
2059	MIRU 20	77	TCGGAGAGATGCCCTTCGAGTTAG (FAM)<br>GGAGACCGCGACCAGGTACTTGTA
2687	MIRU 24	54	CGACCAAGATGTGCAGGAATACAT<br>GGGCGAGTTGAGCTCACAGAA (VIC)
3007	MIRU27	53	TCGAAAGCCTCTGCGTGCCAGTAA<br>GCGATGTGAGCGTGCCACTCAA (NED)

Tab1e S2. Mycobacterial Interspersed Repetitive Units – Variable Number Tandem Repeats (MIRU-VNTR) 12 loci data.

ID	Well	0580	0802	2996	0960	3192	1644	0154	4348	2531	2687	2059	3007
1	A01	2	2	1		3		2	2	5	1	2	3
2	B01	3	3					2		5			
3	C01	3	3	5	4	3	3	2	2	5	1	2	3
4	D01	2	1	6		5		2	3	5	1	2	1
5	E01	2	1			4		2	3	5	1	2	3
6	F01	2	1	5	3	3		2	2	8	1	2	2
7	G01	2	1	7	3	5		2	3	5	1	2	1
8	H01	2	4	5	2	2	3	1	2	6	1	2	3
9	A02	2	4	5		2		1	2	6	1	2	3
10	B02	2	1	5	4	3	1	2	2	6	1	2	2
11	C02											2	3
12	D02	3	3									2	
13	E02	2	3	7	3	4	4	2	2	5	1	2	3
14	F02	3	3	4	4	3	3	2	2	5	1	2	3
15	G02												
16	H02	2	1					2					
17	A03	2	2					2	2	5	1	2	3
18	B03	3	3	5	4	3		2	2	5	1	2	3
19	C03												
20	D03	2	3	7		4		2		5	1	2	3
21	E03	2	1	7	3	5	3	2	3	5	1	2	1
22	F03	2	1	7	3	5	3	2	3	5	1	2	1
23	G03	2	1	7	3	5	3	2	3	5	1	2	1
24	H03	2	1	7	3	5	3	2	3	5	1	2	1
25	A04	2	1	7	3	5	3	2	3	5	1	2	1
26	B04	2	1	7	3	5	3	2	3	5	1	2	1
27	C04	5	3	2	4	5	3	2	1	4	2	2	3
28	D04	2	1	7	3	5	3	2	3	5	1	2	1
29	E04	2	1	7	3	5	3	2	3	5	1	2	1
30	F04	5s	5	2	4	6	3	2	2	7	2	2	3
31	G04	2	1	7	3	5	3	2	3	5	1	2	1
32	H04	2	1	7	3	5		2	3	5	1	2	1
33	A05	2	1									2	3
34	B05	2	4	1	7	4	4	2	3	5	1	2	3
35	C05	2	1	4	4	2	3	1	2	6	1	2	3
36	D05	3	3	5	4	3	3	2	2	5	1	2	3
37	E05	2	1	7	3	5	3	2	3	5	1	2	1
38	F05	2	1	8	3	5	3	2	3	5	1	2	1
39	G05	2	1	4	4	3	1	2	2	6	1	2	2
40	H05	2	3	7	3	4	4	2	2	5	1	2	3

Note: MIRU-VNTR 12 loci (coded Green) generated in the study. ID is the sample number. Raws with pink codes are un-amplifiable regions

Table S3. ***Mycobacterium tuberculosis*** **complex strains isolated from all the samples.**

Sample Number	*M. tuberculosis* strain
1	Mixed Match (URAL, Harleem, TUR)
2	Unknown
3	Mixed Match (Cameroon, Harleem)
4	Unknown
5	Mixed Match (Ghana, Delhi/CAS)
6	Unknown
7	Unknown
8	LAM
9	LAM
10	LAM
11	Bovis
12	Unknown
13	Uganda I
14	Unknown
15	Unknown
16	Mixed Match (Uganda II, West African I & II)
17	Uganda I
18	Unknown
19	Unknown
20	Beijing
21	Beijing
22	Beijing
23	Beijing
24	Beijing
25	Beijing
26	Beijing
27	EAI
28	Beijing
29	Beijing
30	Unknown
31	Beijing
32	Unknown
33	Mixed Match (West African I & II)
34	Unknown
35	LAM
36	Mixed Match (Cameroon, Harleem)
37	Beijing
38	Unknown
39	LAM
40	Uganda I
